# Comparison of the Genetic Basis of Yield Traits Between Main and Ratoon Rice in an Eight-Way MAGIC Population

**DOI:** 10.3390/plants14223527

**Published:** 2025-11-19

**Authors:** Zhongmin Han, Ahmed Sherif, Mohammed Ayaad, Yongzhong Xing, Yuncai Lu

**Affiliations:** 1 College of Advanced Agriculture and Ecological Environment, Heilongjiang University, Haerbin 150006, China; hanzm_vp@163.com; 2 National Key Laboratory of Crop Genetic Improvement and National Center of Plant Gene Research (Wuhan), Huazhong Agricultural University, Wuhan 430070, China; ahmed_elsaid_sherif@agr.kfs.edu.eg (A.S.); mohnad_ayaad@yahoo.com (M.A.); yzxing@mail.hzau.edu.cn (Y.X.); 3 Rice Research Department, Field Crops Research Institute, Agriculture Research Center, Sakha 33717, Egypt; 4 Plant Research Department, Nuclear Research Center, Atomic Energy Authority, Abo-Zaabal 13759, Egypt

**Keywords:** ratoon rice, yield, ratooning ability, tillering, GWAS, QTL

## Abstract

Ratoon rice plays a crucial role in sustainable rice production due to its potential for additional harvests; however, the genetic basis of its yield remains to be explored. In this study, we aimed to precisely dissect the genetic basis of yield in ratoon rice by selecting 302 eight-way MAGIC lines that achieved synchronized heading within a 10-day period through staggered sowing. The eight parental lines exhibited distinct yield performances across both main and ratoon crops. Significant correlations were observed between the main and ratoon crops concerning panicle length (R = 0.67) and spikelets per panicle (R = 0.36). Genome-wide association studies (GWAS) revealed a total of 17 quantitative trait loci (QTLs) associated with five yield-related traits in both main and ratoon crops. Specifically, seven QTLs were detected for yield components in the main crop, while six QTLs were identified in the ratoon crop, in addition to five QTLs associated with ratooning ability. Notably, only one QTL, *qPL1,* was commonly detected in both crops, exhibiting opposite effects on tiller number across crop types. Among the QTLs specifically identified in the ratoon crop, *qGY10* demonstrated the largest effect on ratoon grain yield without compromising the performance of the main crop. The known gene, *Ghd7.1*, exhibited pleiotropic effects on both ratooning ability and ratoon grain yield. Candidate gene analysis prioritized likely causal genes and defined key haplotypes within these QTL intervals by leveraging the genomic diversity of the eight founders. These findings underscore the distinct genetic determinants for yields in main and ratoon crops, providing a genetic basis for breeding high-yielding varieties in both crop types.

## 1. Introduction

Ratoon rice refers to the subsequent generation of rice that grows from the axillary buds on the stubble left behind after the main crop has been harvested. Typically, ratoon rice yields 40–50% of the main crop’s production and is recognized for its superior appearance and cooking characteristics [1,2]. Furthermore, the inputs required for ratoon rice cultivation are significantly reduced; fields do not need to be re-prepared, and the application of fertilizers and pesticides is substantially decreased [3]. Consequently, ratoon rice cultivation has been widely adopted as a cost-effective method, particularly in Asia.

The Tiller Number (TN) is closely related to ratoon yield, which is significantly influenced by ratooning ability (RA) [4]. RA refers to the potential for regenerated tillers to emerge from dormant buds on the stubble, and it is currently the primary focus in this study of ratoon yield. Various factors affect RA, including genetic components, environmental conditions, and management practices; consequently, the TN of ratoon crops is typically not highly correlated with that of the main crop [5,6]. Several studies have identified quantitative trait loci (QTLs) associated with RA. For example, *qRAT5* was detected in a BC_5_F_3_ population; and is closely linked to a QTL for spikelets per panicle (SPP) [7]. However, the effects of *qRAT5* on TN and grain yield per plant (GY) have not been documented. Additionally, *qRA5* was identified within a 530-kb region through an introgression line population [5], influencing the TN ratio and GY ratio of ratoon crops relative to the main crop. *qRA2* was fine-mapped to a 233-kb region using near-isogenic lines [8], and it modestly increased RA, seed setting rate and yield per plant. The major QTLs identified in these studies for RA colocalized with other yield-related traits, raising the question of whether gene clusters or pleiotropic genes are involved. *RRA3*,which encodes a nucleoredoxin protein, was cloned from a rice collection and is known to negatively regulate RA [9]. The introgression of a weak expression allele of *RRA3* into Guichao 2 resulted in a 23.8% increase in RA and ratoon yield. This gene exhibited significant differentiation between *indica* and *japonica* varieties, with haplotype 1, which has the highest RA, present in many *indica* but absent in *japonica* accessions.

Besides TN, panicle length (PL) and spikelet per panicle (SPP) in ratoon rice plants are crucial for enhancing ratoon rice grain yield (GY) [10,11]. Both PL and SPP can be improved through field management practices such as nitrogen application and stubble height adjustments. While SPP significantly influences the yield of the main crop, it has been reported that a high number of SPP in the main crop negatively affects ratoon yield due to competition for photosynthetic resources [12]. This competition can be alleviated by increasing nitrogen application in the ratoon crop to support tiller bud development. However, the direct effects of SPP and PL in the main crop on their corresponding traits in the ratoon crop have rarely been studied. Thus, it remains unclear whether the genetic basis for ratoon crop yield differs from that of the main crop.

Most studies on the genetic basis of ratoon rice have been conducted using bi-parental populations such as recombinant inbred line (RIL), introgression line (IL), near-isogenic line (NIL), and backcross (BC) populations [5,7,8], with few studies utilizing a germplasm collection [9]. Bi-parental populations derived from two diverse parents are powerful for mapping QTLs due to their suitability for linkage analysis [13,14]. However, only a limited number of QTLs for a target trait are detected in a single bi-parental population, and QTL effects are estimated between two parental alleles. In contrast, natural populations exhibit high genetic diversity and extensive historical recombination events, enabling genome-wide association studies (GWAS) to map more QTLs with higher resolution [15,16]. Nevertheless, natural populations are subject to strong population structure, which can lead to false-positive QTLs unless properly controlled through principal component analysis or kinship considerations. To address this issue, some studies have conducted separate GWAS for *indica* and *japonica* populations [17,18].

Multiparent advanced generation intercross (MAGIC) populations are established through pairwise crosses among multiple parents, followed by several generations of self-crossing. This design offers key advantages for QTL mapping: it achieves high genetic diversity and balanced allele frequency by equally sampling from multiple founders; it minimizes spurious population structure through repeated intercrossing; and it enhances mapping resolution via abundant recombination events that break down linkage disequilibrium [19,20,21]. These attributes render MAGIC populations particularly valuable for unraveling the genetic basis of complex traits, such as grain yield (GY). In this study, we performed gene-based and SNP-based GWAS to identify QTLs associated with root architecture (RA), and yield-related traits in both the main crop and the ratoon crop of a MAGIC population. We also compared the genetic foundations of related traits between the ratoon and main crops. The favorable alleles of identified QTLs are recommended to breeders for enhancing the yield of ratoon rice.

## 2. Results

### 2.1. Parental Performance of the MAGIC Population in Main and Ratoon Seasons

In the main season, Cypress exhibited the highest grain yield of 51.0 g, which correlated with a high number of spikelets per panicle. GC2, IR34, and ZS97 demonstrated moderate GY values of 31.0 g, 30.1 g, and 37.3 g, respectively (Table S1). Notably, GC2 displayed a high SPP of 307.4, while IR34 had a high TN of 16 and a long PL of 26.3 cm. Conversely, MH63 and YJSM recorded the lowest GY values of 9.3 g and 7.0 g, respectively. MH63 was characterized by a low SPP of 120.3, and YJSM was identified as late-maturing, both factors contributing to their diminished GY. Pratao and DA5 did not flower throughout the entire growth season in Wuhan, China; therefore, phenotypic data for these two varieties were unavailable.

In the ratoon season, IR34 and YJSM showed the highest GY values of 26.1 g and 26.4 g, respectively. IR34 exhibited a high SPP of 119.4, whereas YJSM exhibited strong RA of 1.6 and a high TN of 23. GC2 and MH63 presented moderate GY values of 14.1 g and 10.3 g, respectively. GC2 maintained a high SPP of 307.4, while MH63 showcased a high RA of 1.8 and a TN of 28.5. ZS97 and Cypress recorded the lowest GY values of 1.7 g and 4.3 g, respectively. ZS97 showed relatively low values for all traits associated with grain production. Despite Cypress having a strong RA of 1.6, it had a limited number of tillers in the ratoon crop (12.0), which may be attributed to its low TN in the main crop (7.5).

### 2.2. Yield Performance of the MAGIC Population in Main and Ratoon Crops

**Figure 1:**
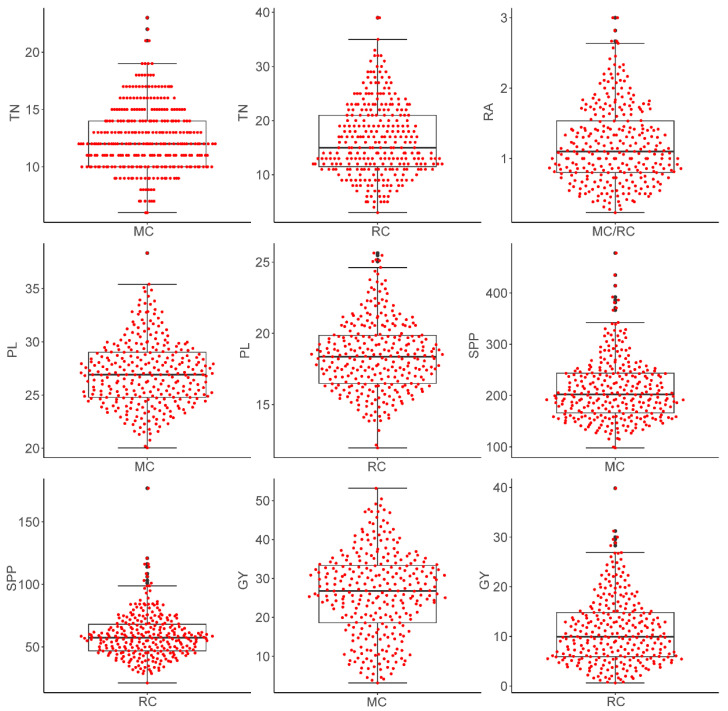
The phenotype distribution of rice ratooning ability and yield related traits of the main and ratoon crop. TN, tiller number; SPP, spikelets per panicle; PL, panicle length; GY, grain yield. MC, main crop; RC, ratoon crop. Most yield-related traits exhibited lower values in RC than in MC, whereas TN increased markedly in the ratoon crop, reflecting its strong regenerative tillering potential.

Through staggered sowing in three batches, heading was synchronized across all lines within a 10-day period. In the main crop, TN ranged from 6.0 to 23.0 with an average of 12.6 among the 302 MAGIC lines (Figure 1 and Figure S2). Plant length (PL) ranged from 20.0 to 38.3 cm with an average of 27.1 cm. SPP ranged from 97.9 to 477.7 with an average of 213.2. GY ranged from 3.2 g to 53.2 g with an average of 25.8 g. The grain yield of the main crop in this population was primarily influenced by TN (β = 0.25, *p* < 0.001) and, to a lesser extent, by SPP (β = 0.15, *p* < 0.01, Figure S3). The ratoons headed 25 to 40 days after harvesting the main crop. For ratoon crop, TN exhibited a wider range, spanning from 3.0 to 39.0, with an average of 16.4 (Figure 1 and Figure S4). RA ranged from 0.23 to 3.0 with an average of 1.2. Ratoon PL was generally shorter than that of the main crop, ranging from 12.0 to 25.6 cm with an average of 18.4 cm. The SPP of the ratoon crop was generally lower than that of the main crop, ranging from 21.2 to 176.0 with an average of 59.3. Consequently, ratoon GY ranged from 0.63 g to 39.8 g with an average of 11.0 g. The grain yield of the ratoon crop was primarily influenced by TN (β = 0.52, *p* < 0.001), SPP (β = 0.29, *p* < 0.001) and PL (β = 0.13, *p* < 0.01, Figure S3).

Across the two seasons, the correlation coefficients of the same traits varied between environments: PL showed a high correlation of 0.67, SPP displayed a moderate correlation of 0.36, while TN (R = 0.21) and GY (R = −0.13, Figure S4) showed no significant correlations. Notably, 67% of the lines demonstrated a greater number of tillers in the ratoon crop compared to the main crops. This observation indicates that ratooning ability plays a crucial role in determining ratoon TN in addition to the TN of the main crop. Two-way ANOVA revealed that genotype (G), environment (E), and the G × E interaction significantly contributed to the phenotypic variation observed for TN, PL, SPP, and GY (Table S2). The seasonal conditions during the main and ratoon cropping periods represented the largest component of variation for PN, SPP and GY. For instance, PL exhibited a substantial contribution from genotype (24.5%) but also had the highest environmental contribution (69.0%) to phenotypic variation, resulting in a heritability estimate of 0.25. Conversely, TN displayed the smallest environmental contribution (8.4%), alongside the largest contributions from genotype (45.7%) and G × E interaction (34.1%), leading to a higher heritability estimate (*h*^2^ = 0.51).

### 2.3. The Genetic Architecture of Yield and Ratooning Ability Across Cropping Seasons

**Figure 2:**
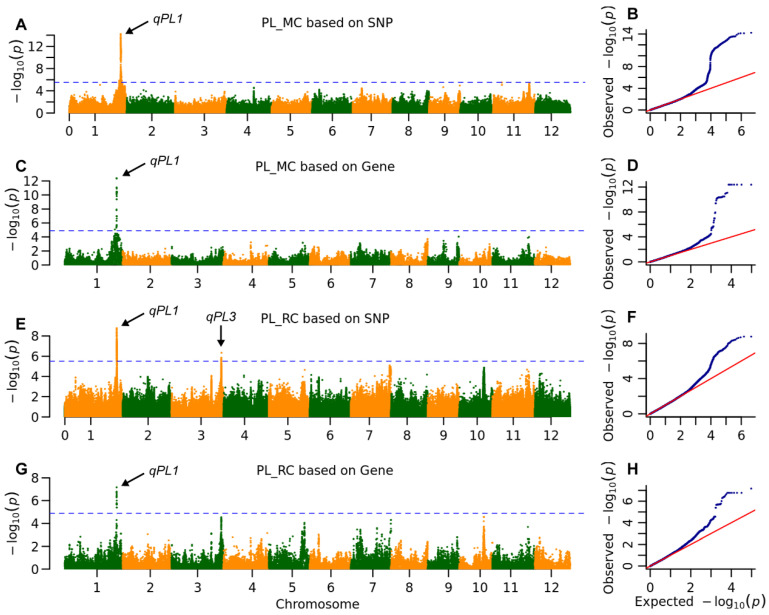
The whole genome association signals of panicle length (PL). Manhattan plot of the association signals for (**A**) main crop (MC) based on SNP, (**C**) main crop based on gene, (**E**) ratoon crop (RC) based on SNP and (**G**) ratoon crop based on gene, the horizontal blue line indicates the threshold of 3.1 × 10^−6^ and 1.4 × 10^−5^ for SNP-base GWAS and gene-based GWAS, respectively. Arrows highlight significant association signals identified on chromosomes 1 and 3, pointing to genetic loci governing panicle length. (**B**,**D**,**F**,**H**) Q-Q plot of association signals for (**A**,**C**,**E**,**G**). **The blue dots represent the observed -log10(*****P*****-values), and the red line indicates the expected null distribution.** The close adherence of the data points to the diagonal line indicates that the mixed linear model effectively controlled for population structure. The upward deviation of the tail ends confirms the presence of true genetic associations beyond what would be expected by chance.

**Figure 3:**
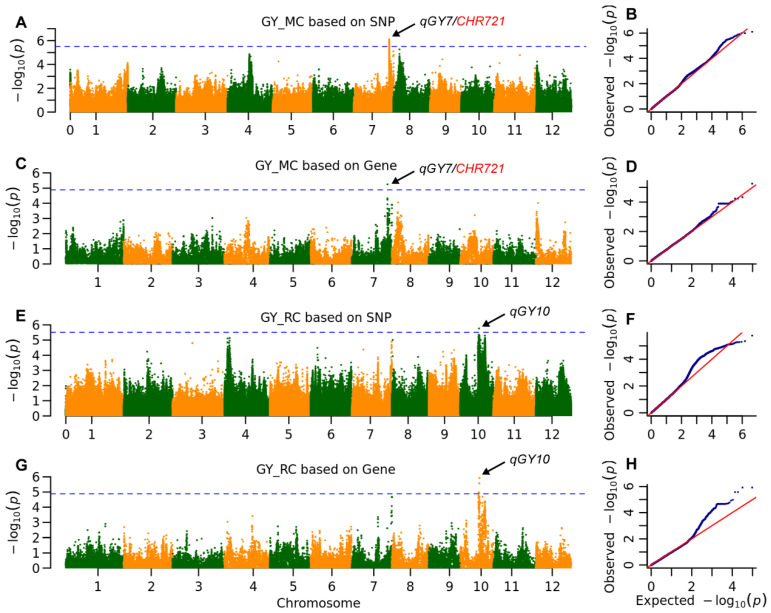
The whole genome association signals of grain yield (GY). Manhattan plot of the association signals for (**A**) main crop (MC) based on SNP, (**C**) main crop based on gene, (**E**) ratoon crop (RC) based on SNP and (**G**) ratoon crop based on gene, the horizontal blue line indicates the threshold of 3.1 × 10^−6^ and 1.4 × 10^−5^ for SNP-base GWAS and gene-based GWAS, respectively. Arrows highlight significant association signals identified on chromosomes 7 and 10, pointing to genetic loci governing grain yield. (**B**,**D**,**F**,**H**) Q-Q plot of association signals for (**A**,**C**,**E**,**G**). **The blue dots represent the observed -log10(*****P*****-values), and the red line indicates the expected null distribution.** The close adherence of the data points to the diagonal line indicates that the mixed linear model effectively controlled for population structure. The upward deviation of the tail ends confirms the presence of true genetic associations beyond what would be expected by chance.

**Figure 4:**
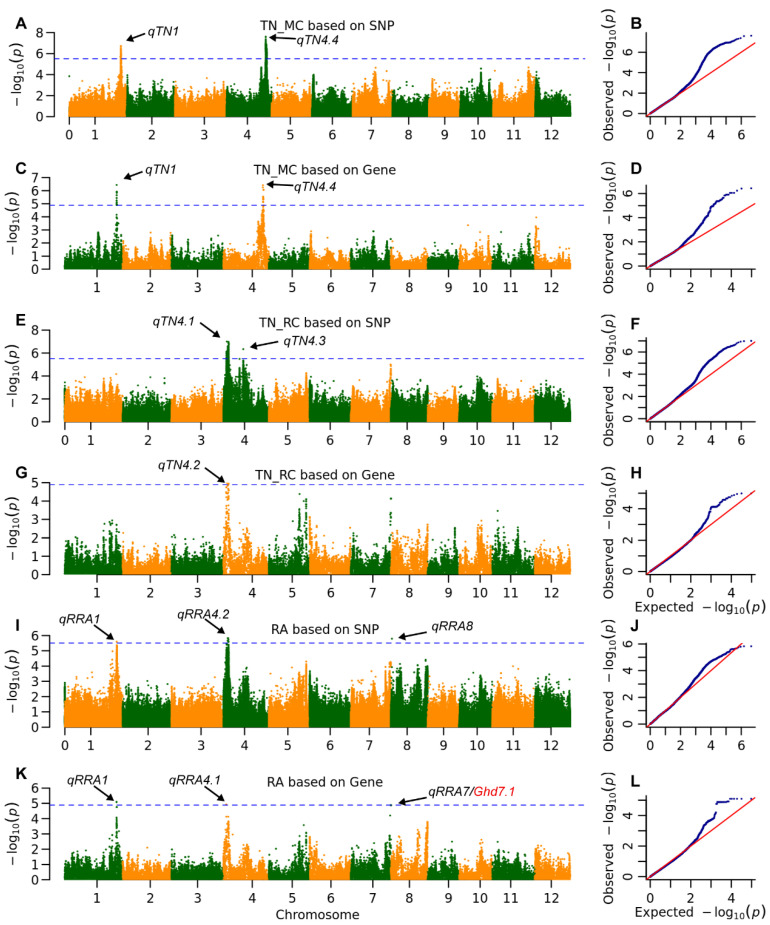
The whole genome association signals of tiller number (TN) and ratooning ability (RA). ACEG). Manhattan plots of genome-wide association signals for TN, based on (**A**) SNP-based GWAS, (**C**) gene-based GWAS in the main crop (MC), and (**E**) SNP-based GWAS, (**G**) gene-based GWAS in the ratoon crop (RC). Arrows indicated key loci on chromosomes 1 and 4 governing tiller number. (**B**,**D**,**F**,**H**) Corresponding Q-Q plots for (**A**,**C**,**E**,**G**) illustrating the distribution of observed versus expected *p*-values. The horizontal blue lines indicate significance thresholds of 3.1 × 10^−6^ for SNP-based GWAS and 1.4 × 10^−5^ for gene-based GWAS. (**I**,**K**) Manhattan plots of genome-wide association signals for RA, based on (**I**) SNP-based GWAS and (**K**) gene-based GWAS. Arrows indicated key loci on chromosomes 1, 4, 7 and 8 governing ratooning ability. (**J**,**L**) Corresponding Q-Q plots for RA. **The blue dots represent the observed -log10(*****P-*****values), and the red line indicates the expected null distribution.** The close adherence of the data points to the diagonal line indicates that the mixed linear model effectively controlled for population structure. The upward deviation of the tail ends confirms the presence of true genetic associations beyond what would be expected by chance.

A total of 12 QTLs associated with yield-related traits were identified across both growing seasons (see Table 1 and Table 2, Figure 2, Figure 3 and Figure 4 and Figure S5). Two QTLs were specifically identified for PL. The QTL *qPL1* was detected in both the main and ratoon crops through both SNP-based and gene-based GWAS, accounting for over 20% and 10% of the phenotypic variations observed in the main and ratoon crops, respectively. In contrast, *qPL3* was identified solely in the ratoon crop via SNP-based GWAS, explaining 5.8% of the phenotypic variation.

For SPP, three QTLs were detected in the main crop (refer to Figure S5), while none were identified in the ratoon crop. The QTL_S_ *qSPP1* and *qSPP2* were identified through SNP-based GWAS, explaining 10.9% and 7.0% of the phenotypic variation, respectively. Additionally, *qSPP4* was detected by both methods, showing an explanation of 11.2% of the variation according to the SNP-based method and 8.5% according to the gene-based method.

Regarding GY, one QTL was detected in each season: *qGY7* in the main crop and *qGY10* in the ratoon crop (Figure 3, Table 1). The QTL *qGY7* was detected in the main crop and explained 6.7% of the phenotypic variation according to both methods (Table 1 and Table S5). The position of *qGY7*, as identified by gene-based GWAS is located 3.1 kb from *CHR721*, a gene involved in the regulation of both male and female reproductive development [22]. Conversely, *qGY10* was detected in the ratoon crop, explaining 6.8% and 5.1% of the phenotypic variation as assessed by the two methods.

Five QTLs were detected for TN in both seasons (Table 2, Figure 4). Two QTLs, *qTN1* and *qTN4.4*, were identified in the main crop using both mapping methods, accounting for approximately 10% of the phenotypic variation. In the ratoon crop, three QTLs were detected; specifically, *qTN4.1* and *qTN4.3* were mapped using SNP-based GWAS, explaining 11.6% and 4.4% of the phenotypic variation, respectively, while *qTN4.2* was mapped through gene-based GWAS. Notably, no TN QTLs were commonly detected between the main and ratoon crops.

Similarly, five QTLs were detected for rice RA (Table 2, Figure 4). The QTL *qRA1* was identified by both mapping methods, explaining approximately 8% of the phenotypic variation. Both *qRA4.2* and *qRA8* were detected exclusively through SNP-based GWAS, explaining 7.0% and 5.9% of the phenotypic variation, respectively. Conversely, *qRA4.1* and *qRA7* were detected solely through gene-based GWAS, explaining 1.3% and 5.4% of the phenotypic variation, respectively. Importantly, *qRA7* was precisely located at *Ghd7.1*, a gene that significantly influences heading date, plant height, and grain yield [23,24].

### 2.4. Genetic Effects of Multiple Parental Alleles at Key QTLs Impacting Ratoon Crop Performance

Here, we primarily focus on QTLs that influence ratoon crop yield and ratooning ability, examining their effects on both crop types. The QTL *qTN1* is located at ORF MH01t0732900 and encodes an S-locus-like receptor protein kinase (SRK). Its homologous gene, *OsLSK1*, has been reported to affect bud growth by promoting primary branches on the panicle and responding to gibberellin (GA) [25]. Additionally, *OsLSK1* forms heterodimers with other SRK genes. The seven missense variations in MH01t0732900 resulted in three haplotypes (Table 3). Hap2 decreased the number of tillers by 1.7 in the main crop but increased it by 1.9 in the ratoon crop, with its higher RA of 1.4 primarily attributed to the lower TN in the main crop compared to the other two alleles.

The QTL *qPL1* is located at ORF MH01t0733100 and encodes an APETALA2 (AP2)/Ethylene-Responsive Element Binding Factor (ERF). The rice homologous gene of MH01t0733100, *OsEATB*, has been reported to reduce PL by mediating the cross-talk between ethylene and GA [26]. Among the eight parental alleles of MH01t0733100, there is only one missense variation that formed two haplotypes (Table S3). In the main crop, the Hap2 allele increased PL by 2.7 cm, and SPP by 33.3, but reduced the number of tillers by 1.5, resulting in no effect on GY. In contrast, in the ratoon crop, the Hap2 allele increased PL by 1.7 cm, the number of tillers by 2.2, and SPP by 6.5, ultimately leading to an increase of 2.2 g in grain weight in the ratoon crop. The Hap2 allele of *qPL1* has differing impacts on GYs in the two seasons, primarily due to its opposing effects on TNs.

The *qGY10* gene was detected exclusively in the ratoon crop and is associated with the ORF MH10t0253200, which encodes a protein containing pumilio-family RNA binding repeats. Pumilio genes are known to respond to both biotic and abiotic stresses, thereby aiding plants in adapting to fluctuating environmental conditions [27]. The homologous gene of MH10t0253200 in Arabidopsis, *APUM24*, plays a critical role in seed maturation [28]. Overexpression of *APUM24* has been shown to increase seed size and weight. The MH10t0253200 gene exhibits numerous variations, including four frameshift mutations (Table 4) and 20 missense variations (Table S4). These variations have resulted in the formation of four haplotypes among the eight parental lines. Notably, Hap4, identified in Cypress was present in 21 lines and was associated with an increase in TN by 4.5, RA by 0.3 (though this was not statistically significant due to its low allele frequency of 7.5%), PL by 1.9 cm, SPP by 13.0 and GY by 8.6 g. Conversely, Hap3 found in DA5, led to an increase in TN by 2.0 and SPP by 3.9, but resulted in a reduction in PL by 0.5 cm, ultimately increasing GY by 3.3 g.

A single insertion-deletion (InDel) event classified the parental alleles at the *Ghd7.1* locus into two haplotypes (Table 5). The Hap2 allele present in IR34 and ZS97, features an 8-bp deletion in the coding sequence (CDS), resulting in a loss-of-function mutation. In contrast, the Hap1 allele found in GC2, DA5, Cypress, YJSM, MH63 and Protao, was associated with increases in TN by 3, RA by 0.3, SPP by 4.8 and GY by 3.5 g in the ratoon crop.

## 3. Discussion

### 3.1. Diverse Genetic Basis of Grain Yield Between Main and Ratoon Crops

In the main crop, TN contributed the most significantly to grain yield among the three investigated traits (Figure S3). No lines exhibited the highest TN and SPP simultaneously (Figure S2), likely due to a compensatory mechanism between these two factors [29]. Both TN and SPP significantly contribute to ratoon grain yield (Table 3, Table 5 and Table S3, Figure S4). Typically, the average grain yield of the population in the ratoon crop is lower than that of the main crop, primarily due to a significantly reduced SPP in ratoons (Figure S4). Consequently, for each line, PL in the ratoon crop was shorter than that in the main crop (Table S3). The tillers in the main crop, which originate from the basal unelongated internodes require a longer time to develop panicles, whereas the tillers in the ratoon rice arising from the elongated internodes, complete panicle development in a shorter duration. This discrepancy likely explains why PL in the ratoon crop is considerably shorter than that in the main crop.

A significant correlation was observed only in PL and SPP between the main and ratoon crops, while no correlation was found between the two environments for TN and grain yield (Figure S4). These results indicated that the genetic basis of grain yield likely differs between the main and ratoon crops. Furthermore, QTL mapping revealed that seven and six QTLs were detected for four traits in the main and ratoon crops, respectively. Notably, only one QTL, *qPL1,* was identified in both crop types (Figure 2, Table 1). These findings clearly demonstrate a diverse genetic basis for grain yield between the two types of crops. In the ratoon crop, Hap2 of *qPL1* positively influences all traits contributing to grain yield. Conversely, in the main crop, Hap2 has a negative effect on TN and grain yield, but positive effects on other traits. For large-scale rice production, Hap2 can be utilized for high-density planting in the main crop due to its low TN and the increase in ratoon crop yield attributed to its positive impact on ratooning tillers. Among the QTLs detected in single crop type, *qGY10* exhibited the largest effect on grain yield in the ratoon crop but no effect in the main crop. This specific QTL is particularly valuable for enhancing ratoon yield without negatively impacting the grain yield of the main crop.

### 3.2. Genetic Insights into Tiller Number and Ratooning Ability

TN of the ratoon crop often surpasses that of the main crop, significantly contributing to the ratoon yield [30]. The TN of the ratoon is influenced by both the TN of the main crop and the RA [31]. Since the number of dormant buds in the stubbles is fixed post-harvest of the main crop, the RA becomes a crucial factor in regulating both ratoon TN and overall grain yield. Tiller bud formation occurs at each leaf axil during the development of the main crop. Tiller buds on basal unelongated internodes predominantly give rise to most tillers, whereas those on elongated internodes are largely suppressed [32]. The latter buds remain dormant until the main crop is harvested [2]. Consequently, the number of tillers in the ratoon is likely greater than that in the main crop, as more than one dormant tiller bud per main tiller has the potential to develop (Figure S4).

Five QTLs for TN were identified in the MAGIC population, none of which were detected across both seasons (Table 2, Figure 4). Previously reported key tillering genes *MOC1* [32] and *OsTB1* [33] were not detected in this study. Further analysis revealed no variations in the coding sequences of these genes among the eight parental lines, indicating that both genes are functional across the eight parents, likely due to artificial selection during the breeding of high-yielding varieties. Five QTLs were detected for RA, but the RA gene *RRA3* was not detected. The Hap1 allele of *RRA3* exhibits stronger RA compared to Hap6, and Hap1 is exclusively found in *indica* varieties rather than *japonica* varieties [9]. In this study, we observed that the only *japonica* parent, Cypress, carries the reported Hap6 allele but exhibits a similar RA (1.1) to the alleles carried by the seven *indica* parents (Table S6). This may be attributed to the relatively minor effect of *RRA3* within the genetic background of this MAGIC population.

Several novel QTLs and beneficial alleles have been identified for RA and GY. The beneficial alleles of *qRA7*/*Ghd7.1* and *qGY10* both enhance RA by 0.3, which is associated with an increase in grain yield (see Table 4 and Table 5). Notably, the favorable allele of *qGY10* originated from the cypress parent, which exhibited a lower tiller count (7.5) in the main crop but a higher RA (1.6, see Table 4, and Supplementary Table S1). This observation leads us to speculate that the introduction of a high RA allele from a variety with low main crop TN into another variety with a high TN in the main crop could be a promising strategy for effectively improving the TN of the ratoon crop. Therefore, future high-yielding ratoon cultivars should be characterized by optimal TN in the main crop and strong RA through the pyramiding of QTLs for TN in the main crop and RA.

### 3.3. Hormones Might Be the Major Endogenous Factor Regulating Ratooning Ability in Ratoon Crop

The dormancy of axillary buds is primarily maintained by apical dominance. Indole-3-acetic acid (IAA) released from the shoot apical meristem (SAM) inhibits axillary bud outgrowth by downregulating cytokinin (CK) levels and upregulating strigolactone (SL) content in the axillary bud [34]. SL inhibits axillary bud outgrowth and acts within the bud itself by depleting PIN1 auxin efflux proteins, which are essential for the formation of the polar auxin transport system [35]. The inhibitory effect of SL on axillary bud outgrowth is dependent on OsTB1 although SL does not regulate its transcription. MOC1 is protected from degradation by the DELLA protein SLENDER RICE 1 (SLR1). Gibberellins (GAs) trigger the degradation of SLR1, which subsequently leads to the degradation of MOC1, resulting in stem elongation and a decrease in TN [36]. It is likely that these genes involved in hormone synthesis or signaling are associated with RA. In our study, two QTLs affecting ratoon TN were identified: *qPL1* and *qTN1*. Both may participate in GA signaling because their homologous genes, *OsLSK1* and *OsEATB*, are known to respond to and mediate GA signals [25,26]. The QTL *qGY10* exclusively promotes ratoon TN and yield. its candidate encodes a pumilio family protein that has been reported to respond to auxin and cytokinin [27]. Further research is needed to uncover the mechanisms of hormone regulation in ratoon tillers.

### 3.4. The Challenge of QTL Mapping for Ratooning Ability in Ratoon Crop

In addition to genetic components, the outgrowth of dormant tiller buds is influenced by environmental factors such as temperature, disease occurrence, and insect activity. Synchronizing the heading date of a mapping population is essential for accurately estimating RA. In this study, all MAGIC lines flowered within a 10-day window due to staggered sowing, and all lines were harvested within a short period, which minimized discrepancies in the physiological status of dormant buds in the stubbles. RA is also influenced by stubble height [37]. Harvesting the main crop removes apical dominance from the stubble, thereby releasing dormancy in buds from the upper to the lower nodes. A lower cutting height (<20 cm) during the main crop harvest is generally recommended to increase grain yield, as more panicles are generated from basal nodes [30,38]. However, it is important to note that a lower cutting height often extends the growth duration of the ratoon crop, necessitating an assessment of whether the ratoon crop can mature within the growing season of specific ecological regions. To ensure that all ratoons can mature, uniform 30-cm stubbles were maintained after the harvest of the main crops, despite the MAGIC lines exhibiting diverse plant height in the main crop. This cutting method likely results in stubbles of MAGIC lines bearing dormant tiller buds with varying physiological statuses, thereby diminishing the effectiveness of QTL mapping for RA. Addressing this challenge of maintaining uniform conditions for ratooning growth will enhance the power of QTL mapping.

## 4. Materials and Methods

### 4.1. Development of a MAGIC Population

Eight parents were selected to construct an 8-way MAGIC population. Among these, Cypress is the sole japonica variety, originating from America and known for its early maturity. The remaining seven parents are indica varieties: Minghui63 (MH63) and Zhenshan 97 (ZS97), which are the parental lines of the elite Chinese hybrid Shanyou 63; the high-yield variety Guichao 2 (GC2); the high-quality variety Yuejingsimiao (YJSM); the cold-tolerance variety Pratao (Pra); as well as IR34 and DA5. The MAGIC population was established through three rounds of hybridization (Figure S1). In the first round, hybridizations were conducted between DA5 and Pra, ZS97 and MH63, YJSM and IR34, and GC2 and Cypress, resulting in four two-way F_1_ hybrids. In the second round, two 4-way hybrid families were created by crossing the hybrid DA5/Pra with the hybrid ZS97/MH63, and the hybrid YJSM/IR34 with the hybrid GC2/Cypress. In the third round, 144 8-way F_1_ families were generated through pairwise crossing of the two four-way F_1_ families. Four plants from each 8-way F_1_ family were selected in the F2 generation. Subsequently, the single-seed descent strategy was implemented over the next five generations. Ultimately, 560 MAGIC lines were obtained in the F_7_ generation.

### 4.2. Field Management and Phenotype Measurement

To minimize environmental noise for ratoon rice, 302 MAGIC RILs that reached the heading stage within a concentrated one-month window were selected for this study. To synchronize the heading dates, these 302 RILs were divided into three batches based on their respective heading dates, with staggered sowing occurring on 25 March, 5 April, and 15 April 2019, in Wuhan, China. Ten 25-day-old seedlings from each MAGIC line were transplanted into the fields with two replicates. The distance between plants in a row was set at 16.5 cm, while the distance between rows was maintained at 26.4 cm. The main crops were fertilized 10 days prior to harvesting, with all lines harvested around 10 August. Tillers of the main crop were trimmed, leaving a 30-cm stubble for ratooning. Standard rice ratooning practices were adhered to for field management. The measured traits included TN, PL, SPP, and GY for both the main and ratoon crops. RA was calculated as the TN ratio of the ratoon crop to the main crop.

### 4.3. Next-Generation Sequencing and Genotyping

DNA was extracted from the leaves of MAGIC lines using the CTAB method. DNA libraries were constructed using the TD501 toolkit from the TruePrep DNA Library Prep Kit V2 for Illumina. Sequencing was performed on a HiSeq 3000 System with a depth of 30× for the parental samples and 5× for the MAGIC lines.

Reads from the 302 MAGIC lines were aligned to the reference genome Minghui63 (http://rice.hzau.edu.cn/rice_rs1/; **accessed on 15 October 2025**) using BWA [39]. SAM files were sorted by coordinate and PCR duplicates were marked using the SortSAM and MarkDuplicates tools available in Picard software 2.23.3 (http://broadinstitute.github.io/picard/; **accessed on 8 October 2025**). Variants were called using the HaplotypeCaller function from the GATK toolkit [40], and were filtered based on the following parameters: QualbyDepth > 1, Mapping quality > 40, and Fisher Strand < 80. Variants with a minor allele frequency (MAF) < 0.05, a missing rate < 0.8, a heterozygous rate < 0.05, and a mean depth > 2 and <15 were retained. This process yielded 2,625,079 high-quality variation sites.

### 4.4. Genome-Wide Association Studies

To exploit the abundant recombination events and high genetic diversity in MAGIC population, we constructed the haplotypes of each annotated gene on the reference genome from variations (SNPs and INDELs) in its coding regions. Then, both SNP-based and gene-based GWAS were performed using a mixed linear model (MLM):

y = Xβ + Zu + Wγ + e

where *y* is a vector of phenotypic observations, *β* represents the fixed effects of SNPs or gene haplotypes, *X* is an incidence matrix relating *y* to *β* constructed using dummy variables for SNPs or genes), *u* is a vector of polygene background effects with $u\sim N(0,K\sigma_{G}^{2})$, where *K* is a matrix of relative kinship coefficients calculated from nucleotide diversities, and $\sigma_{G}^{2}$ is the genetic variance. *e* is a vector of residual effects with $e\sim N(0,I\sigma_{e}^{2})$, where I is an identity matrix and $\sigma_{e}^{2}$ is the residual variance. Wγ accounts for fixed effects of sowing batch and other environmental covariates. The SNP-based model was implemented in FastLMM software v2.07.20140723 [41], while the gene haplotype model was executed using the “rrBLUP” package [42] in R software 4.4.2. GWAS thresholds were determined using improved Bonferroni methods based on the effective number of independent tests. For SNP-based GWAS, the effective number was calculated to be 15,948, resulting in a significance threshold of $P<\frac{0.05}{15948}=3.1\times 10^{-6}$. For Gene-based GWAS, the effective number was calculated to be 3693, resulting in a significance threshold of $P<\frac{0.05}{3693}=1.4\times 10^{-5}$.

### 4.5. Genetic Statistics

Correlation coefficients between different traits were calculated using the cor() function in R software. The plot_model() function of sjPlot package in R software was used to analyze the contributions of TN, SPP and PL to grain yield. Two-way ANOVA was performed using the lm() and anova() functions in R software to assess the contributions of genotype, environment, and their interactions, with environments including main and ratoon seasons. The explained phenotypic variation was analyzed using the lm() function in R, where peak SNPs or gene haplotypes from GWAS were treated as independent variables. Multiple comparisons for each candidate gene were conducted using the duncan.test() function in the agricolae package (1.3.7) of R software. The heritability was calculated by the formula: $h^{2}=V_{g}/V_{p}$, where $V_{g}$ is the genotypic variance, $V_{p}$ is the phenotypic variance [43].

## 5. Conclusions

Significant phenotypic variation was observed between the main and ratoon crops. The identification of season-specific QTLs not only underscores the complexity of yield determination across cropping seasons but also provides critical genetic resources for breeding high-yielding rice varieties. By pyramiding favorable alleles for key traits, such as main crop TN and RA, it is possible to develop cultivars that optimize productivity in both seasons.

## Figures and Tables

**Table 1:** The detected yield-related QTLs for main crop and ratoon crop of rice.

Trait	Crop	QTL	SNP-Based GWAS			Gene-Based GWAS				Nearby Gene
			Pos	*p*	PV (%)	Peak Gene	Pos (bp)	*p*	PV (%)	
PL	MC	*qPL1*	36,766,995	5.8 × 10^−15^	19.5	MH01t0732300	36,790,627	4.3 × 10^−13^	24.0	
	RC	*qPL1*	36,746,675	1.7 × 10^−9^	11.1	MH01t0733100	36,818,061	3.8 × 10^−9^	10.7	
		*qPL3*	35,566,823	4.5 × 10^−7^	5.8					
SPP	MC	*qSPP1*	36,912,397	2.3 × 10^−8^	10.9					
		*qSPP2*	2,890,022	1.8 × 10^−6^	7.0					
		*qSPP4*	28,118,380	1.9 × 10^−7^	11.2	MH04t0625100	28,096,106	1.3 × 10^−5^	8.5	
GY	MC	*qGY7*	25,360,255	7.8 × 10^−7^	6.7	MH07t0500600	25,371,872	5.6 × 10^−6^	6.7	3.1 kb to *CHR721*
	RC	*qGY10*	13,346,352	1.7 × 10^−6^	6.8	MH10t0253200	13,694,183	1.2 × 10^−6^	5.1	

SPP, spikelets per panicle; PL, panicle length; GY, grain yield. MC, main crop; RC, ratoon crop.

**Table 2:** The detected rice ratooning ability-related QTLs.

Trait	Crop	QTL	SNP-Based GWAS			Gene-Based GWAS				Nearby Gene
			Pos	*p*	PV (%)	Peak Gene	Position (bp)	*p*	PV (%)	
TN	MC	*qTN1*	36,895,057	1.9 × 10^−7^	7.8	MH01t0732900	36,812,637	3.6 × 10^−7^	10.1	
		*qTN4.4*	28,100,407	2.4 × 10^−8^	10.6	MH04t0625200	28,101,376	3.8 × 10^−7^	8.9	
	RC	*qTN4.1*	2,333,293	9.9 × 10^−8^	11.6					
		*qTN4.2*				MH04t0067200	2,745,892	1.0 × 10^−5^	2.1	
		*qTN4.3*	14,036,939	4.6 × 10^−7^	4.4					
RA		*qRRA1*	36,742,004	2.5 × 10^−6^	8.3	MH01t0731300	36,742,004	8.1 × 10^−6^	7.4	
		*qRRA4.1*				MH04t0054700	2,082,223	1.2 × 10^−5^	1.3	
		*qRRA4.2*	3,268,530	1.5 × 10^−6^	7.0					
		*qRRA7*				MH07t0551600	28,514,126	1.3 × 10^−5^	5.4	*Ghd7.1*
		*qRRA8*	664,886	1.6 × 10^−6^	5.9					

TN, tiller number; RA, ratooning ability. MC, main crop; RC, ratoon crop.

**Table 3:** The variations in and haplotype effects of MH01t0732900—the peak gene of *qTN1*.

	Hap 1	Hap 2	Hap 3
G1212C (Lys404Asn)	G	C	G
T1440C (Cys480Cys)	T	C	T
C1962T (Cys654Cys)	C	T	C
C2030T (Ala677Val)	C	T	C
T2104C (Ser702Pro)	T	C	C
A2201G (As34Gly)	A	G	A
G2227T (Ala743Ser)	G	T	G
G2365C (Val789Leu)	G	C	G
G2385T (Glu795Asp)	G	T	G
Frequency	168	95	22
Parents	IR34/GC2/Cyp/ YJMS/MH63	DA5/Pra	ZS97
TN_MC	13.2 ± 3.1 b*	11.5 ± 2.2 c	14.5 ± 3.5 a
TN_RC	15.5 ± 6.1 a	17.4 ± 7.4 a	16.6 ± 7.1 a
RA	1.1 ± 0.5 b	1.4 ± 0.6 a	1.0 ± 0.4 b
PL_MC	26.3 ± 2.7 b	28.8 ± 3 a	24.9 ± 3.2 c
PL_RC	18 ± 2.3 b	19.5 ± 2.6 a	17 ± 2.7 b
GY_MC	26.2 ± 10.3 a	24.4 ± 11.9 a	27.9 ± 12.5 a
GY_RC	10.4 ± 6.3 a	12.2 ± 6.9 a	10.4 ± 6.8 a

* The phenotype values are represented as the means ± SD, and the letters indicated significant differences (*p* < 0.05 by Duncan test).

**Table 4:** The variations in and haplotype effects of MH10t0253200—the peak gene of *qGY10*.

	Hap 1	Hap 2	Hap 3	Hap 4
Ins 2222–2223 (FS)	0	0	0	1
Del 2219 (FS)	0	0	0	−1
Ins 2192–2193 (FS)	0	0	0	2
Del 2190–2191 (FS)	0	0	0	−2
Frequency	168	72	19	21
Parents	IR34/MH63/ Pra/ZS97	GC2/YJMS	DA5	Cyp
TN_MC	12.6 ± 2.7 a*	12.2 ± 3.3 a	12.4 ± 2.5 a	12.9 ± 2.9 a
TN_RC	16.1 ± 6.5 b	15.4 ± 6.2 b	17.4 ± 8.9 a,b	19.7 ± 8.3 a
RA	1.2 ± 0.6 a	1.1 ± 0.5 a	1.3 ± 0.7 a	1.4 ± 0.5 a
PL_MC	27.1 ± 3.1 a	27.0 ± 3.1 a	26.8 ± 3.1 a	28.2 ± 3.5 a
PL_RC	18.4 ± 2.4 b	18.2 ± 2.8 b	17.7 ± 2.4 b	20.1 ± 2.3 a
SPP_MC	217.7 ± 68.3 a	215.3 ± 63.3 a	196.8 ± 32.0 a	218.6 ± 64.7 a
SPP_RC	59.9 ± 18.7 a,b	55.5 ± 17.6 b	59.4 ± 14.8 a,b	68.5 ± 13.4 a
GY_MC	25.6 ± 10.6 a	25.9 ± 12.2 a	26.3 ± 10.1 a	25.7 ± 12.6 a
GY_RC	10.7 ± 6.2 bc	9.4 ± 5.7 c	12.7 ± 8.2 b	18.0 ± 9.1 a

* The phenotype values are represented as the means ± SD, and the letters indicated significant differences (*p* < 0.05 by Duncan test). Only four frame shift variations among the 24 variations were exhibited.

**Table 5:** The variations in and haplotype effects of *Ghd7.1*.

	Hap 1	Hap 2
Del 1515_1522; (FS *)	0	−8
Frequency	168	124
Parents	GC2/DA5/Cyp/ YJSM/MH63/Pra	IR34/ZS97
TN_RC	17.7 ± 7.1 a	14.7 ± 6 b
RA	1.3 ± 0.6 a	1.0 ± 0.5 b
PL_MC	27.4 ± 3.3 a	26.7 ± 2.9 b
PL_RC	18.8 ± 2.5 a	17.9 ± 2.4 b
SPP_MC	225.4 ± 68.1 a	196.2 ± 54.3 b
SPP_RC	61.3 ± 18.1 a	56.5 ± 18.3 b
GY_MC	24.2 ± 11 b	27.4 ± 10.8 a
GY_RC	12.5 ± 7.1 a	9.0 ± 5.5 b

The phenotype values are represented as the means ± SD, and the letters indicated significant differences (*p* < 0.05 by Duncan test). * FS means Frameshift mutation.

## Data Availability

The next-generation sequencing data for the 302 MAGIC lines and 8 parents have been deposited in the NCBI Sequence Read Archive (SRA) database under the BioProject accession number PRJNA1217574.

## Supplementary Materials

The following supporting information can be downloaded at https://www.mdpi.com/article/10.3390/plants14223527/s1, Figure S1: The construction diagram of eight-way MAGIC population; Figure S2: The correlationship of tiller number and spikelets per panicle in main season; Figure S3: Divergent genetic effects of yield-related traits on grain yield in the main and ratoon crops; Figure S4: The correlationship of yield-related traits between two seasons; Figure S5: The whole genome association signals of spikelet per panicle; Table S1: The performance of MAGIC parents in main and ratoon crops; Table S2: Genotypes and environments analysis of variance for yield-related traits using 302 MAGIC lines; Table S3: The variations and haplotype effects of MH01t0733100—the peak gene of *qPL1*; Table S4: The variations and haplotype effects of MH10t0253200, the peak gene of *qGY10*; Table S5: The variations and haplotype effects of MH07t0500600—the adjacent gene of *qGY7*; Table S6: The variations and haplotype effects of *RRA3*.
