# Supplementary material for: Comparison of the Genetic Basis of Yield Traits Between Main and Ratoon Rice in an Eight-Way MAGIC Population
Source: Plants (Basel). 2025 Nov 19;14(22):3527. doi: 10.3390/plants14223527 (PMC12656013; doi:10.3390/plants14223527)
Supplement: Supplementary file 1 [file plants-14-03527-s001.zip › plants-3932793-supplementary.pdf]

Table S1. The performance of MAGIC parents in main and ratoon crops

	CYP	GC2	IR34	MH63	YJSM	ZS97
TN_MC	7.5 ± 0.7	11.5 ± 0.7	16.0 ± 1.4	16.0 ± 1.4	14.5 ± 2.1	12.5 ± 2.1
TN_RC	12.0 ± 4.2	17.5 ± 6.4	11.5 ± 3.5	28.5 ± 0.7	23.0 ± 8.5	4.5 ± 0.7
RA	1.6 ± 0.4	1.5 ± 0.6	0.7 ± 0.2	1.8 ± 0.1	1.6 ± 0.4	0.4 ± 0.01
PL_MC	25.6 ± 0.4	25.4 ± 1.0	26.3± 0.1	27.6 ± 0.8	23.9 ± 1.9	22.4 ± 0.4
PL_RC	15.9 ± 0.7	17.3 ± 1.1	13.6 ± 0.2	19.9 ± 0.2	17.4 ± 0.4	15.0 ± 0.1
SPP_MC	363.9 ± 82.4	307.4 ± 8.6	214.8 ± 10.9	120.3 ± 0.08	179.3 ± 5.7	172.5 ± 7.2
SPP_RC	41.4 ± 20.6	104.8 ± 5.0	119.4 ± 4.3	30.9 ± 11.0	82.5 ± 37.2	42.4 ± 13.0
GY_MC	51.0 ± 7.7	31.0 ± 14.8	30.1 ± 0.6	9.3 ± 0.8	7.0 ± 3.1	37.3 ± 5.1
GY_RC	4.3 ± 0.9	14.1 ± 7.7	26.1 ± 7.8	10.3 ± 5.6	26.4 ± 1.7	1.7 ± 0.2

The phenotypes of Prato and DA5 was not presented because they didn't flower during the entire growth season in Wuhan.

Table S2. Genotypes and environments analysis of variance for yield-related traits using 302 MAGIC lines

Trait	Resources	Df	SSE/SST (%)	F	*p*
TN	G	301	45.7	7.7	<0.0001
	E	1	8.4	423.9	<0.0001
	G × E	295	34.1	5.8	<0.0001
	Error	598	11.8		
PL	G	301	24.5	20.9	<0.0001
	E	1	69.0	17731.1	<0.0001
	G × E	295	4.2	3.6	<0.0001
	Error	598	2.3		
SPP	G	301	16.6	8.7	<0.0001
	E	1	69.9	11074.6	<0.0001
	G × E	292	9.8	5.3	<0.0001
	Error	588	3,7		
GY	G	300	24.3	6.0	<0.0001
	E	1	37.3	2773.9	<0.0001
	G × E	293	30.6	7.8	<0.0001
	Error	588	7.9		

Table S3. The variations and haplotype effects of MH01t0733100-the peak gene of *qPL1*

	Hap 1	Hap 2
A6T (Ala2Ala)	A	T
T182A (Val61Glu)	T	A
Frequency	194	102
Parents	IR34/Guichao2/Cypress/Yuejingsimiao/Minghui63/Zhenshan97	DA/Pratao
TN_MC	13.1 ± 3.0 a	11.6 ± 2.3 b
TN_RC	15.6 ± 6.2 b	17.8 ± 7.4 a
RA	1.1 ± 0.5 b	1.4 ± 0.6 a
PL_MC	26.1 ± 2.7 b	28.8 ± 3.0 a
PL_RC	17.8 ± 2.3 b	19.5 ± 2.5 a
SPP_MC	201.9 ± 58.2 b	235.2 ± 69.4 a
SPP_RC	57.0 ± 15.8 b	63.5 ± 21.2 a
GY_MC	26.4 ± 10.8 a	24.6 ± 11.6 a
GY_RC	10.3 ± 6.3 b	12.5 ± 7.5 a

*The phenotype values are represented as the means ± SD, and the letters indicated significant differences ($P < 0.05$ by Duncan test).

Table S4. The variations and haplotype effects of MH10t0253200, the peak gene of *qGY10*

	Hap 1	Hap 2	Hap 3	Hap 4
C145A (Leu49Ile)	C	C	C	A
G234A (Ser78Ser)	G	G	G	A
G301A (Gly101Ser)	G	G	G	A
C303T (Gly101Gly)	C	C	C	T
C304T (Arg102Cys)	C	C	C	T
G393A (Thr131Thr)	G	G	G	A
G397T (Val133Phe)	G	G	G	T
A451T (Asn151Tyr)	A	A	A	T
C501T (Cys167Cys)	C	C	C	T
T544G (Ser182Ala)	T	T	T	G
T552C (As84As)	T	T	T	C
A577G (Ile193Val)	A	A	A	G
C579T (Ile193Ile)	C	C	C	T
G919A (Gly307Ser)	G	G	G	A
G930A (Ser310Ser)	G	A	A	A
T968G (Phe323Cys)	T	T	T	G
A1035G (Ala345Ala)	A	A	A	G

C1037G (Thr346Ser)	C	C	C	G
C1131T (Ile337Ile)	C	C	C	T
T1173C (Cys392Arg)	T	T	T	C
T1314G (Ala438Ala)	T	T	T	G
T1329C (His443His)	T	T	T	C
A1446G (Ala482Ala)	A	A	A	G
T1485C (Leu495Leu)	T	T	T	C
C1800T (Ala600Ala)	C	C	C	T
C1959T (Thr653Thr)	C	C	C	T
T2010A (Val670Val)	T	T	T	A
C2073G (His691Gln)	C	C	C	G
G2155T (Ala719Ser)	G	G	G	T
C2187T (As29As)	C	C	C	T
2090-2191 (DelAG)	TGA	TGA	TGA	T
2192-2193 (DelAC)	T	T	T	TCA
C2205A (Ala735Ala)	C	C	C	A
2219 (DelC)	AC	AC	AC	A
G2222A (Gly741As)	G	G	A	G
2222-2223 (InsA)	T	T	T	TA
C2273G (Ser759Cys)	C	C	C	G
A2289C (Arg763Ser)	A	A	A	C
T2295G (Asn765Lys)	T	T	T	G
A2308G (Lys770Glu)	A	A	A	G
C2316T (Ser772Ser)	C	C	C	T
G2321C (Ser774Thr)	G	G	G	C
A2396G (Lys799Arg)	A	A	A	G
C2433T (Ser811Ser)	C	C	C	A
Frequency	168	72	19	21
Parents	IR34/MH63/Pra/ZS97	GC2/YJMS	DA5	Cyp
TN_RC	16.1 ± 6.5^{b}	15.4 ± 6.2^{b}	17.4 ± 8.9^{ab}	19.7 ± 8.3^{a}
PL_RC	18.4 ± 2.4^{b}	18.2 ± 2.8^{b}	17.7 ± 2.4^{b}	20.1 ± 2.3^{a}
SPP_RC	59.9 ± 18.7^{ab}	55.5 ± 17.6^{b}	59.4 ± 14.8^{ab}	68.5 ± 13.4^{a}
GY_RC	10.7 ± 6.2^{bc}	9.4 ± 5.7^{c}	12.7 ± 8.2^{b}	18 ± 9.1^{a}

[*]The phenotype values are represented as the means ± SD, and the letters indicated significant differences ($P < 0.05$ by Duncan test).

Table S5. The variations and haplotype effects of MH07t0500600-the adjacent gene of *qGY7*

DNA (AA) variation	Hap 1	Hap 2	Hap 3
G321T (Pro107Pro)	G	G	T
G354C (Arg118Ser)	G	G	C
Ins381+1 (Splice_donor_variant)	G	G	GGTTAGGCC
T425C (Leu142Ser)	T	T	C
G774A (Pro258Pro)	A	G	G
C1959T (As53As)	C	C	T
Frequency	144	103	88
Parents	IR34/GC2/YJMS/ZS97	DA5/Cyp	MH63/Pra
GY_MC	28.4 ± 10.5[a]	23.1 ± 10.9[b]	24.3 ± 11.5[b]

[*]The phenotype values are represented as the means ± SD, and the letters indicated significant differences ($P < 0.05$ by Duncan test).

Table S6. The variations and haplotype effects of *RRA3*

	Type 1	Type 2	Type 3	Type 4
G318A (Ser106Ser)	G	A	A	A
G570C (Thr190Thr)	G	G	C	C
T701C (Val234Ala)	T	C	C	C
G944C (Gly315Ala)	G	C	G	G
G1347A (Ser449Ser)	G	G	G	A
A1483G (Asn495Asp)	A	A	A	G
-473-471 Ins TC	T	TTC	TTC	TTC
-416 A>G	A	G	G	G
Frequency	142	9	32	8
Parents	IR34/Guichao2/DA5/Minghui63	Cypress	Yuejingsimiao/Zhenshan97	Pratao
TN_MC	12.4 ± 2.9[a]	12.9 ± 2.3[a]	12.2 ± 2.6[a]	11.8 ± 2[a]
TN_RC	16.3 ± 7[a]	16.4 ± 6.6[a]	15.4 ± 6.5[a]	15.5 ± 7.7[a]
RA	1.2 ± 0.6[a]	1.1 ± 0.4[a]	1.2 ± 0.5[a]	1.2 ± 0.7[a]

[*]The phenotype values are represented as the means ± SD, and the letters indicated significant differences ($P < 0.05$ by Duncan test).

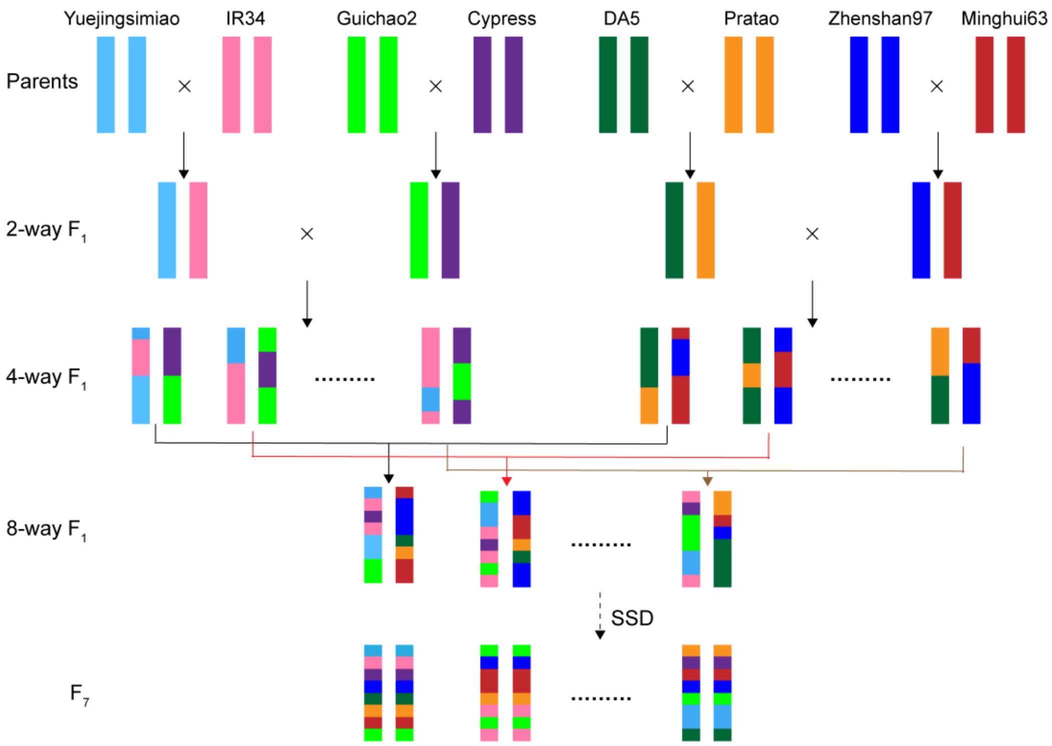

Figure S1. The construction diagram of eight-way MAGIC population

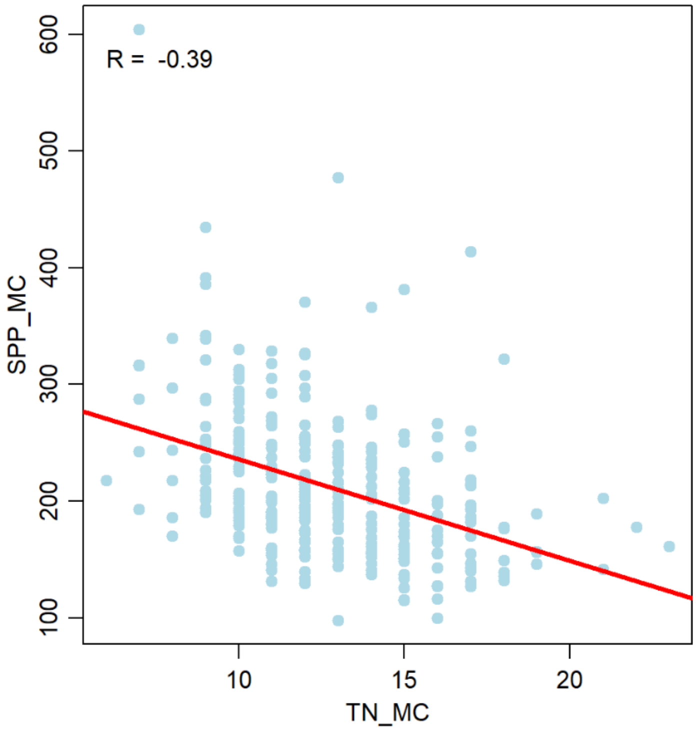

Figure S2. The correlationship of tiller number and spikelets per panicle in main season

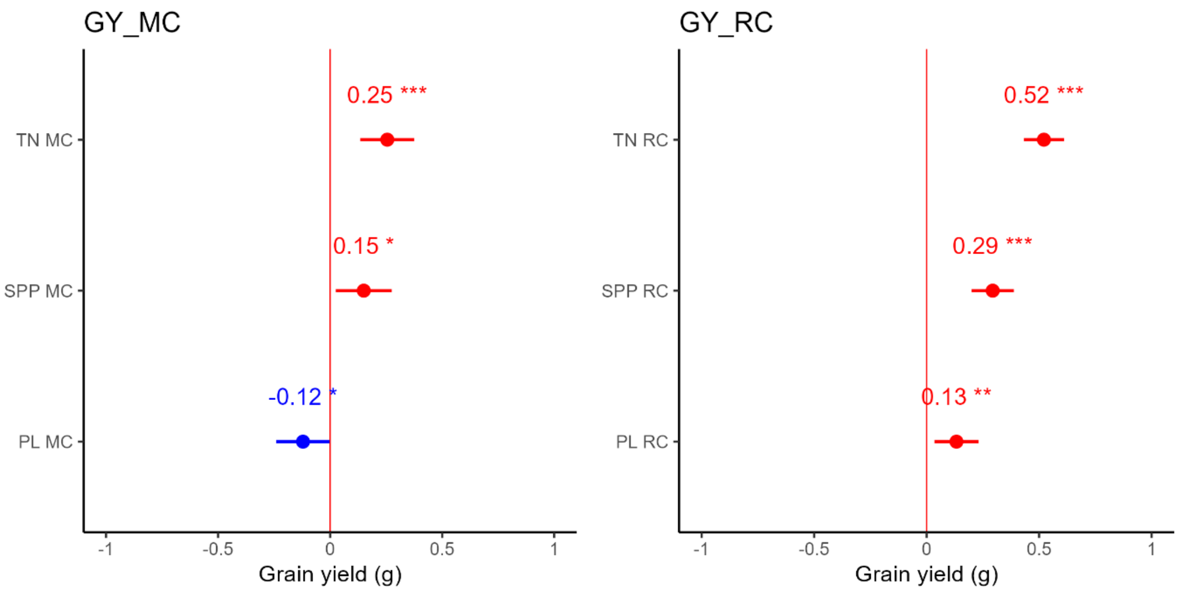

Figure S3. Divergent genetic effects of yield-related traits on grain yield in the main and ratoon crops. Error bars represent 95% confidence intervals. *$P < 0.05$, **$P < 0.001$, ***$P < 0.001$. TN, tiller number; SPP, spikelets per panicle; PL, panicle length; GY, grain yield. MC, main crop; RC, ratoon crop.

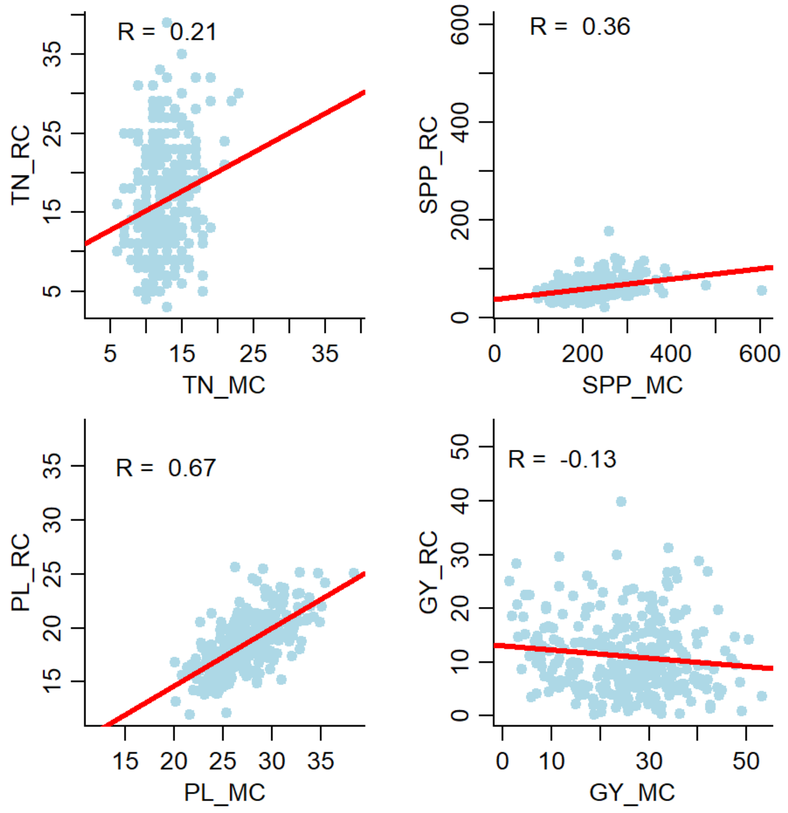

Figure S4. The correlationship of yield-related traits between two seasons. TN, tiller number; SPP, spikelets per panicle; PL, panicle length; GY, grain yield. MC, main crop; RC, ratoon crop.

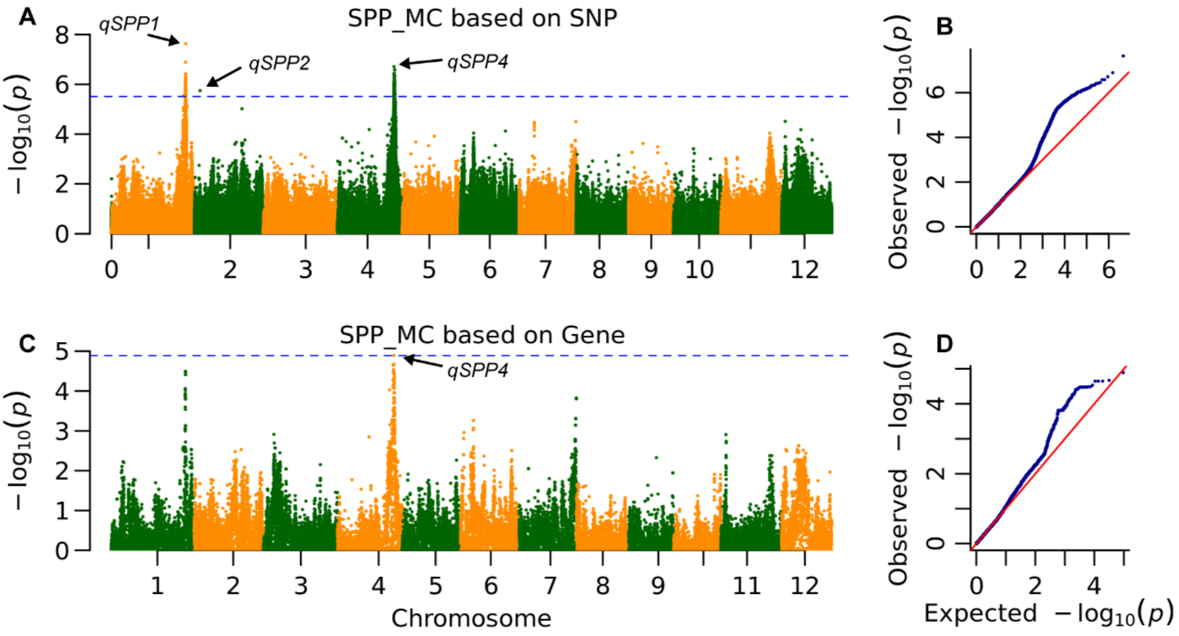

Figure S5. The whole genome association signals of spikelet per panicle. Manhattan plot of the association signals for **a)** main crop based on SNP, **c)** main crop based on gene, the horizontal blue line indicates the threshold of 3.1E-6 and 1.4E-5 for SNP-base GWAS and gene-based GWAS, respectively. **bd)** Q-Q plot of association signals. SPP, spikelets per panicle. MC, main crop.
